# Heterochromatic marks are associated with the repression of secondary metabolism clusters in *Aspergillus nidulans*

**DOI:** 10.1111/j.1365-2958.2010.07051.x

**Published:** 2010-02-01

**Authors:** Yazmid Reyes-Dominguez, Jin Woo Bok, Harald Berger, E Keats Shwab, Asjad Basheer, Andreas Gallmetzer, Claudio Scazzocchio, Nancy Keller, Joseph Strauss

**Affiliations:** 1 Fungal Genetics and Genomics Unit, Austrian Institute of Technology (AIT) and University of Natural Resources and Applied Life Sciences (BOKU) ViennaAustria; 2 Institute de Génétique et Microbiologie, Université Paris-SudUMR 8621 CNRS, Orsay, France; 3 Department of Plant Pathology, Department of Bacteriology and Department of Medical Microbiology and ImmunologyUW-Madison, Madison, WI 53706, USA

## Abstract

Fungal secondary metabolites are important bioactive compounds but the conditions leading to expression of most of the putative secondary metabolism (SM) genes predicted by fungal genomics are unknown. Here we describe a novel mechanism involved in SM-gene regulation based on the finding that, in *Aspergillus nidulans*, mutants lacking components involved in heterochromatin formation show de-repression of genes involved in biosynthesis of sterigmatocystin (ST), penicillin and terrequinone A. During the active growth phase, the silent ST gene cluster is marked by histone H3 lysine 9 trimethylation and contains high levels of the heterochromatin protein-1 (HepA). Upon growth arrest and activation of SM, HepA and trimethylated H3K9 levels decrease concomitantly with increasing levels of acetylated histone H3. SM-specific chromatin modifications are restricted to genes located inside the ST cluster, and constitutive heterochromatic marks persist at loci immediately outside the cluster. LaeA, a global activator of SM clusters in fungi, counteracts the establishment of heterochromatic marks. Thus, one level of regulation of the *A. nidulans* ST cluster employs epigenetic control by H3K9 methylation and HepA binding to establish a repressive chromatin structure and LaeA is involved in reversal of this heterochromatic signature inside the cluster, but not in that of flanking genes.

## Introduction

Fungi are well-known producers of bioactive compounds with antibiotic, growth-regulating, toxic, mutagenic, immunosuppressive, enzyme inhibitory and other biological effects (Keller *et al.*, 2005; Pelaez, 2005). The term ‘secondary metabolism’ (SM) denotes biochemical processes underlying the formation of fungal metabolites such as toxins, antibiotics and immune-suppressing agents (Hoffmeister and Keller, 2007). In the ascomycete fungus *Aspergillus nidulans*, the formation of secondary metabolites is correlated with growth arrest and reproduction (sexual and asexual) and a wealth of information is available on the genetic basis regulating these processes (Calvo *et al.*, 2002; Yu and Keller, 2005). The finding of a nuclear complex including both the light-regulated developmental factor VeA (Calvo *et al.*, 2004; Bayram *et al.*, 2008) and the global regulator of SM, LaeA (Bok and Keller, 2004) establishes a mechanistic link between SM production and morphological differentiation.

Arguably the secondary metabolite synthesis pathway which is best understood is that of sterigmatocystin (ST). Like most other secondary metabolites, the genes required for ST biosynthesis are arranged in a cluster, including the pathway-specific activator gene *aflR* (Keller and Hohn, 1997). A large body of genetic evidence has led to the identification of transcriptional regulators and signalling pathways involved in expression of the ST cluster genes (reviewed in Yu and Keller, 2005). One additional activator of ST production is LaeA. Bok and Keller (2004) showed that LaeA is a nuclear protein required for efficient transcription of the ST cluster, including the pathway activator *aflR*. LaeA contains a predicted and a functionally necessary S-adenosyl-methionine (SAM) binding domain (Bok *et al.*, 2006a). Such domains are present in all members of the methylase superfamily (Kozbial and Mushegian, 2005), including histone methyltransferases involved in chromatin modification and epigenetic gene regulation. LaeA is a conserved fungal protein and has been shown to be a global regulator of chemical diversity (Bok *et al.*, 2006a,b; Perrin *et al.*, 2007; Kosalkova *et al.*, 2009), reproduction (Bayram *et al.*, 2008; Kale *et al.*, 2008) and virulence (Bok *et al.*, 2005) in different *Aspergillus* and one *Penicillium* species.

The cluster arrangement of secondary metabolite genes may facilitate a chromatin-based co-regulation mechanism. Deletion of the *A. nidulans* histone deacetylase HdaA or inhibition of other fungal HDACs by trichostatin A leads to over-production of several secondary metabolites (Shwab *et al.*, 2007). Chichewicz and colleagues treated a number of different fungi with HDAC and DNA methyltransferase inhibitors and found clear evidence for enhanced chemical diversity and higher natural product formation (Williams *et al.*, 2008; Fisch *et al.*, 2009). A position effect has been shown to be involved in the expression of some secondary metabolite biosynthetic and regulatory genes. Ectopic expression of *aflR* bypasses the need for LaeA function and the arginine biosynthetic gene *argB* is silenced in a *laeA*Δ strain when placed inside the ST cluster (Bok *et al.*, 2006a). Similar position effects on secondary metabolite gene regulation have been reported for other *Aspergillus* species as well (Chiou *et al.*, 2002; Roze *et al.*, 2007; Smith *et al.*, 2007). Additionally, our laboratories recently showed that histone H3 lysine 4 di- and trimethylation (H3K4me2/me3), a chromatin mark usually associated with active genes, but also required for telomere silencing in *Saccharomyces cerevisiae* (Mueller *et al.*, 2006), regulates SM in *A. nidulans*. A mutant lacking the Bre2 homologous component (termed CclA in *A. nidulans*) of the COMPASS complex expressed cryptic SM gene clusters in *A. nidulans*. Notably, the reduced amounts of H3K4me in some SM gene promoters in *cclA*Δ strains also resulted in low levels of histone H3 lysine 9 trimethylation (H3K9me3), a chromatin mark associated with gene silencing and heterochromatin formation (Bok *et al.*, 2009). Taken together, these results suggest a strong influence of chromatin-mediated regulation on fungal SM and might represent *bona fide* heritable epigenetic information.

In transcriptionally active chromatin, lysines of histones H3 and H4 (including lysine 9 of histone H3, H3K9) are usually acetylated (Li *et al.*, 2007). Heterochromatic domains are transcriptionally silent and characterized by hypoacetylation of lysines in H3 and H4 (Holbert and Marmorstein, 2005). Lack of acetylation enables mono-, di- or tri-methylation of H3K9 (H3K9me) by the histone methyltransferase Su(var)3-9 in *Drosophila* (Taddei *et al.*, 2001), or Clr4, its orthologue, in *Schizosaccharomyces pombe* (Rea *et al.*, 2000; Noma *et al.*, 2001). This histone modification is recognized by one of the principal components of heterochromatin, the heterochromation protein 1 (HP1) (Cryderman *et al.*, 1998; Wang *et al.*, 2000). HP1 recognizes H3K9me through its amino terminal chromodomain that is necessary for both targeting and transcriptional repression (Fanti and Pimpinelli, 2008). Although HP1 is predominantly associated with pericentric heterochromatin, it is also found at many euchromatic sites where it can promote repression in a H3K9 methylation-dependent or independent process. This system has been found to be conserved in numerous organisms, including filamentous ascomycetes, such as *Neurospora crassa*, where it has been shown to direct DNA methylation (Freitag *et al.*, 2004; Lewis *et al.*, 2009).

To address whether a process analogous to developmentally regulated facultative heterochromatin regulates SM in *A. nidulans*, we deleted the HP1 (SWI6 in *S. pombe*) orthologue HepA and the H3K9-specific methylase ClrD (the orthologue of SU(VAR)3-9 in *D. melanogaster* and of Clr4 in *S. pombe*) and demonstrate their loss leads to over-expression of several *A. nidulans* SM genes. Using chromatin immunoprecipitation (ChIP) we demonstrate that repressive histone marks and high levels of HepA are associated with the silent ST cluster and that LaeA plays a role in reversing this heterochromatic state during the onset of SM. This is the first direct experimental support for a model in which secondary metabolite gene clusters are regulated by a metabolically dependent, reversible formation of heterochromatin.

## Results

### The *A. nidulans hepA* gene encodes a homologue of HP1

The unique putative homologue of HP1, *hepA* (gene number AN1905.3), shows a chromatin modifier protein signature, i.e. a 50-amino-acid N-terminal chromo-domain followed by a C-terminal chromo-shadow domain typical for all known HP1 type proteins. *A. nidulans* HepA shows extended homologies to putative heterochromatin proteins (between 60% and 35% identity, results not shown). Budding yeast *S. cerevisiae* does not contain a HP1 protein and the best studied fungal HP1 homologues are the *S. pombe* Swi6 (Ekwall *et al.*, 1996) and Hpo from *N. crassa* (Freitag *et al.*, 2004). HepA shows limited overall identity (26%) to SWI6, but high similarity in the conserved chromo domain (38% identity) and the chromo-shadow domain (42% identity). Deletion of *hepA* does not lead to any evident morphological or physiological phenotype and the mutant strain grows with the same rate and sporulates as the isogenic wild-type strain (Figs S1 and S2). This is in contrast to several other systems studied so far in which HP1 deletions have been shown to strongly affect viability (reviewed in Hiragami and Festenstein, 2005). Mutations in *S. pombe* Swi6 lead to loss of chromosome stability (Allshire *et al.*, 1995; Ekwall *et al.*, 1996) and *hpo* mutations in *N. crassa* show pronounced growth defects (Freitag *et al.*, 2004).

### Deletion of *hepA* leads to upregulation of secondary metabolite genes

**Fig. 1:**
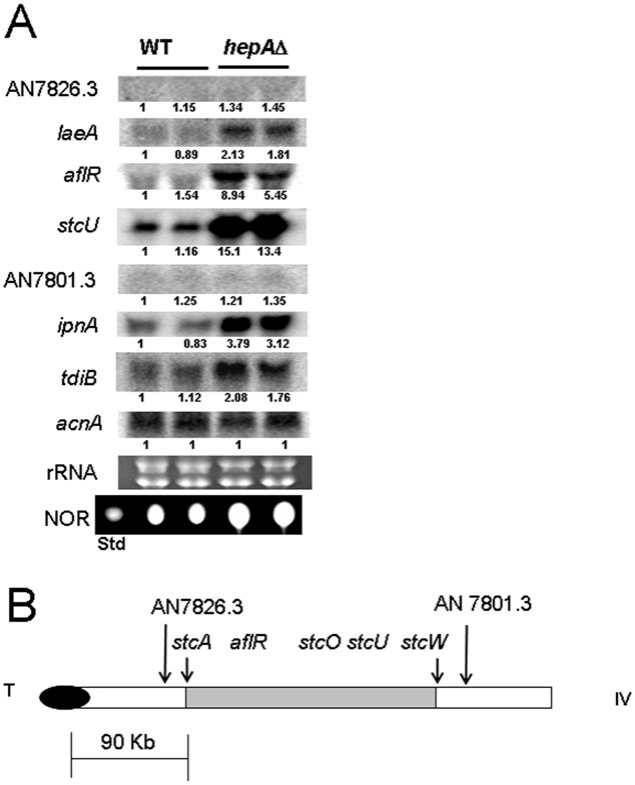
Deletion of *hepA*, the *A. nidulans* HP1 homologue, leads to over-expression of several secondary metabolite genes. A. Comparison of mRNA steady-state levels between *hepA*^+^ wild type (WT) and *hepA*Δ cells cultured in duplicate in liquid GMM for 72 h. The expression levels of *aflR*, and *stcU*, belonging to the sterigmatocystin (ST) gene cluster, together with the genes flanking the ST cluster (AN7826.3 and AN7801.3) are shown. *laeA* encodes the general SM regulator, *ipnA* is a member of the isopenicillin A cluster and *tdiB* is a member of the terraquinone cluster. Actin (*acnA*) and rRNA are shown as loading controls. Numbers below each lane are actin-normalized relative expression levels of the mutant strains in relation to the actin-normalized wild-type control, which has been arbitrarily set to 1. Below the autoradiographs we show a thin-layer chromatography (TLC) plate analysis of the ST precursor norsolorinic acid (NOR; Std, NOR migration standard). B. Overview on the physical map of the ST biosynthetic cluster on chromosome IV. The ST cluster, located roughly 90 kb from the telomere end (T), is represented as shaded box and the approximate position of the two genes delineating the ST cluster (*stcA* and *stcW*) as well as the internal genes mentioned in the text (*aflR*, *stcO*, *stcU*) are shown. Two predicted genes proximal (AN7826.3) and distal (AN7801.3) to the telomere are indicated.

**Fig. 2:**
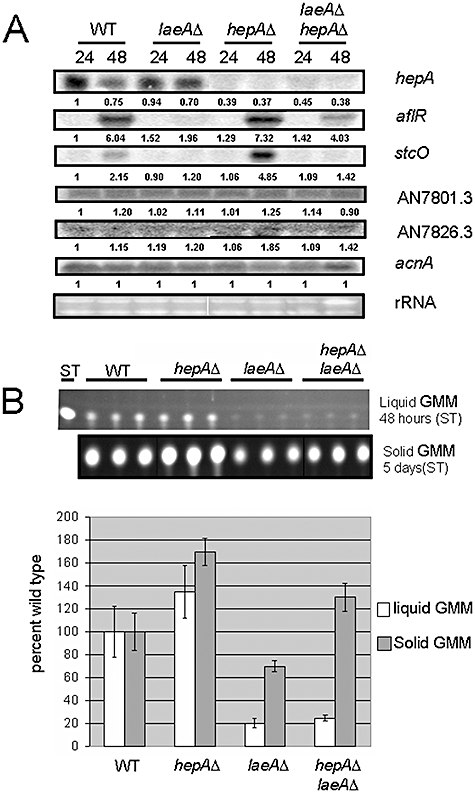
Inactivation of *hepA* leads to enhanced ST gene activation. A. Comparison of mRNA steady-state levels between *hepA*^+^*laeA*^+^ wild type (WT), *laeA*Δ, *hepA*Δ and *laeA*Δ*hepA*Δ cells cultured in GMM for 24 h (active growth, primary metabolism) or 48 h (stationary phase, SM). Two genes of the sterigmatocystin (ST) cluster (*aflR* and *stcO*), the two genes outside the ST cluster (AN7826.3 and AN7801.3) and *hepA* are shown along with the actin (*acnA*) and rRNA loading controls. Numbers below each lane are actin-normalized relative expression levels of the mutant strains in relation to the actin-normalized wild-type control, for which the expression level at 24 h has been arbitrarily set to 1. B. Thin-layer chromatography (TLC) analysis of sterigmatocystin (ST, migration standard) in the wild type, *hepA*Δ, *laeA*Δ and *laeA*Δ*hepA*Δ mutant strains. Three spots per strain represent extracts from three independent GMM liquid cultures grown for 48 h (liquid GMM) or three independent solid cultures grown for 5 days on GMM agar (solid GMM). Densitometry data of TLC plates including standard deviations are represented in the bar graph. WT production levels were assigned a value of 100%, and all other production levels are presented relative to WT. Error bars represent ± one standard deviation.

In a transcriptome analysis comparing submerged cultures of *hepA*Δ to an isogenic wild-type strain we identified several upregulated transcripts coding for proteins involved in SM (data not shown). Northern blots under conditions of secondary metabolite production (for details, see *Experimental procedures*) confirmed the microarray results (Figs 1A and 2A). We tested some genes involved in ST production and found strong overexpression in the *hepA*Δ mutant for the ST-cluster transcription factor *aflR* (∼7-fold), and for two tested structural genes *stcO* (∼2.5-fold) and *stcU* (∼14-fold). Also genes involved in isopenicillin A production (*ipnA*) and in terraquinone A biosynthesis (*tdiB*) were significantly overexpressed. Interestingly, *laeA*, the gene encoding the general regulator of SM in fungi, also shows a ∼2-fold enhanced expression in the *hepA*Δ strain (Fig. 1A and Fig. S3). Importantly, two genes located immediately outside of the ST cluster and expressed invariantly under primary or secondary metabolism conditions (genes AN7826.3 and AN7801.3; Fig. 1B) did not respond to *hepA* deletion suggesting that the effect is restricted to genes located inside the ST cluster. Complementation of the *hepA*Δ strain with the complete *hepA* gene including promoter and terminator sequences resulted in reversal of the *hepA* deletion phenotype, as tested for *aflR* expression (Fig. S4). This demonstrates that the observed *hepA*Δ phenotype was caused by the *hepA* deletion.

Higher *laeA* transcript levels in the *hepA*Δ mutant could be responsible for overexpression of LaeA target genes as observed in expression studies and metabolite analysis (Fig. 1). We therefore tested the genetic interaction between *hepA* and *laeA* and found that in a *hepA*Δ*laeA*Δ double mutant the expression of *aflR,* but not that of *stcO*, is partially remediated in comparison with a *laeA*Δ single mutant (Fig. 2A). As the biosynthesis of ST or its immediate precursor, norsolorinic acid (NOR), requires the expression of all genes in the cluster, we wanted to test if deletion of *hepA* not only remediates *aflR* expression in a *laeA*Δ strain but also leads to remediation of metabolite accumulation. This was not the case. In the *hepA*Δ*laeA*Δ double mutant the partial remediation of *aflR* expression did not translate into remediation of ST production after 48 h of growth in liquid GMM. Unexpectedly, when the strains were grown on solid GMM medium for 5 days, metabolite production in the *hepA*Δ*laeA*Δ double mutant was clearly far above the levels observed in the *laeA*Δ single mutant for ST (Fig. 2B) and also for NOR (Fig. S5A). These data indicate that the higher expression levels of *aflR* caused by the *hepA* deletion leads to slightly increased ST biosynthesis enzyme levels, the activities of which become only apparent as increased metabolite levels after longer incubation periods. Additionally, or alternatively, some environmental factors present only when cultures are grown on solid media allow full restoration of ST production in the absence of LaeA.

### HepA occupancy at the ST cluster decreases during transcriptional activation

**Fig. 3:**
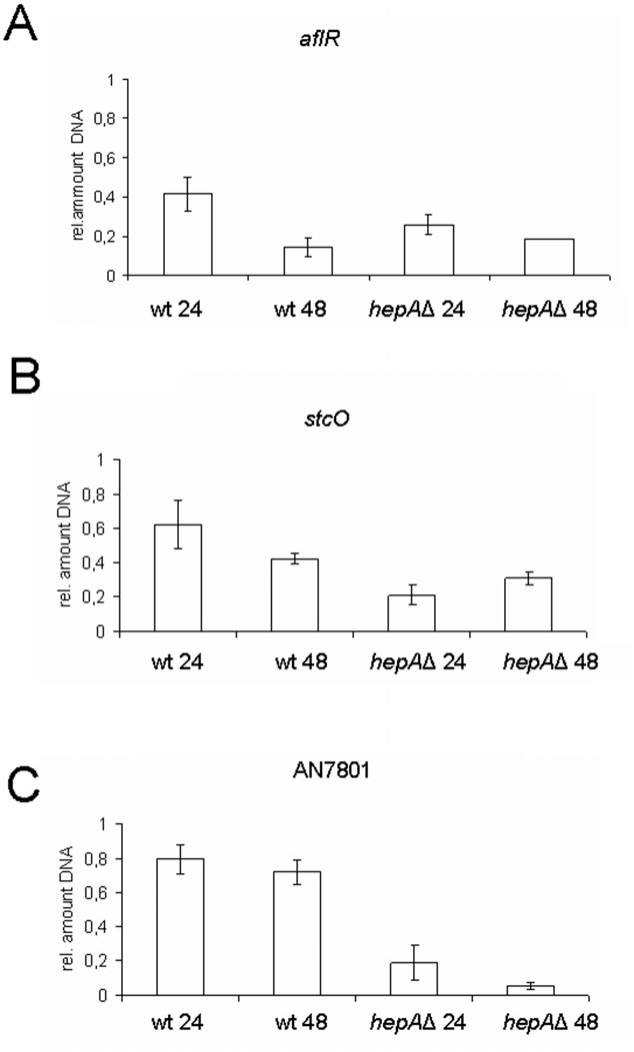
HepA occupancy at different gene promoters and physiological conditions. Wild-type (WT) cells cultured in GMM for 24 h (active growth, primary metabolism) or 48 h (stationary phase, SM) were subjected to HepA ChIP analysis. The gene promoters of two genes (*aflR* and *stcO*, panels A and B respectively) mapping in the sterigmatocystin gene cluster and the promoter of the telomere-distal gene AN7801.3 (panel C) were analysed by quantitative PCR. In each experiment, the *hepA*Δ strain was analysed in parallel to determine the non-specific cross-reaction level of the HP1 antibody used in ChIP. Error bars indicate the standard deviation of two biological and two technical repetitions.

We further tested the role of HepA by measuring HepA occupancy at the ST cluster using ChIP. Because the commercial HP1 antibody considerably cross-reacts with other *A. nidulans* proteins (see Western analysis in Fig. S6) we always performed ChIP reaction in parallel with a *hepA*Δ control strain. Two genes inside the cluster (*aflR, stcO*) and one non-cluster gene were tested at time points when ST genes are not expressed (24 h) or are expressed (48 h). Figure 3A and B show that HepA occupancy at the *aflR* and *stcO* promoter regions significantly decreases in cultures of 48 h as compared with cultures of 24 h. A gene immediately telomere-distal to the ST cluster (locus AN7801) shows high HepA levels and these levels do not change when the neighbouring cluster genes are activated (48 h, Fig. 3C). This suggests that a chromatin boundary element exists between these two coding regions and actively separates the ST cluster from the surrounding heterochromatic region. We investigated the presence of HepA in the bidirectional promoter of the primary metabolism *niiA-niaD* gene cluster in which chromatin remodelling is regulated by different forms of nitrogen (Muro-pastor *et al.*, 1999; Berger *et al.*, 2008). HepA cannot be detected above background levels at this promoter by ChIP at either 24 or 48 h of culture (data not shown). The high HepA levels at the ST locus during the active growth phase hence strongly suggests an important role of heterochromatin in ST gene repression.

### The H3K9 histone methyltransferase *clrD* is involved in ST gene repression

HP1 function requires H3K9me3 to mediate heterochromatin formation. Genome analysis of *A. nidulans* showed one protein (gene number AN1170.3) highly similar to *S. pombe* Clr4 and *N. crassa* DIM-5 (Fig. S7). DIM-5 and H3K9 methylation is required in the latter organism to recruit HP1 as well as DIM-2, a DNA methylase leading to epigenetic silencing (Tamaru *et al.*, 2003; Freitag *et al.*, 2004). In accordance with the *A. nidulans* nomenclature AN1170.3 is called *clrD.* ClrD shares 41% overall identity with the *S. pombe* H3K9 methyltransferase and 38% identity with *N. crassa* DIM-5. ClrD in *A. nidulans* and DIM-5 in *N. crassa* are the only two SET-domain proteins in these organisms showing a predicted pre-SET motif (putative Zn-finger) combined with a SET domain (putative methyltransferase domain). Interestingly, ClrD and the putative orthologues from other filamentous fungi lack the N-terminal chromo-domain found in *S. pombe* Clr4 and other metazoan Su(var)3-9 HMTs (Fig. S7). ClrD is likely the only H3K9 methylase in *A. nidulans* as the deletion strain showed only background signals in Western and ChIP analysis using the H3K9me3-specific antibody (Fig. S8).

**Fig. 4:**
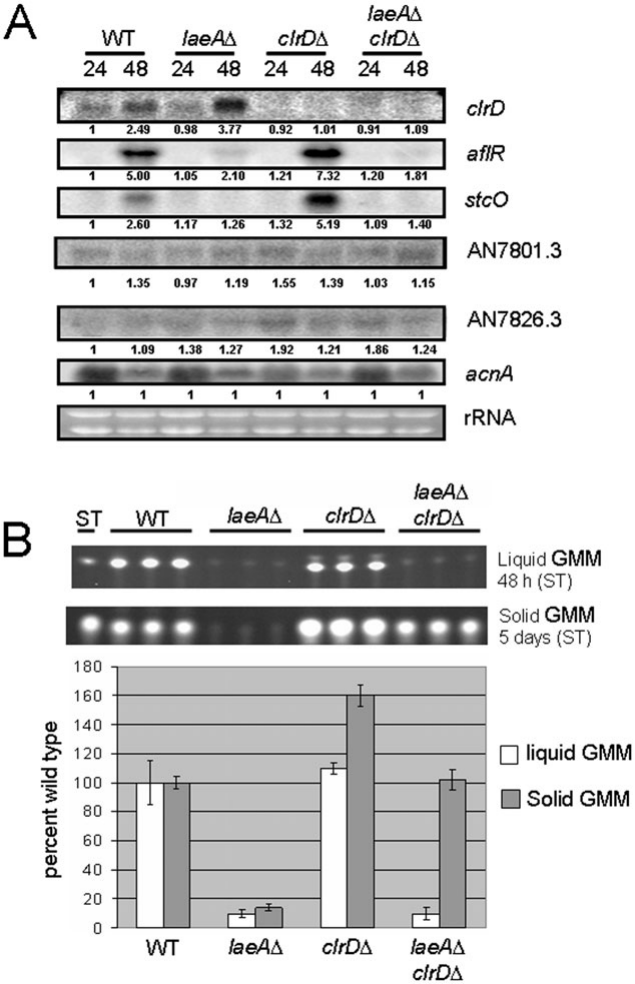
Deletion of *clrD*, the *A. nidulans* histone H3K9 methylase, leads to enhanced ST gene activation. A. Comparison of mRNA steady-state levels between *laeA*^+^*clrD*^+^ wild type (WT), *laeA*Δ, *clrD*Δ and *laeA*Δ*clrD*Δ cells cultured in GMM for 24 h (active growth, primary metabolism) or 48 h (stationary phase, SM). Two genes of the sterigmatocystin (ST) cluster (*aflR* and *stcO*) and the outside genes AN7826.3 and AN7801.3 are shown along with *clrD* and the actin (*acnA*) and rRNA loading controls. Numbers below each lane are actin-normalized relative expression levels of the mutant strains in relation to the actin-normalized wild type control, for which the expression level at 24 h has been arbitrarily set to 1. B. Thin-layer chromatography (TLC) analysis of sterigmatocystin (ST, migration standard) in the wild type, *laeA*Δ, *clrD*Δ and *laeA*Δ*clrD*Δ double mutant strains. Three spots per strain represent extracts from three independent GMM liquid cultures grown for 48 h (liquid GMM) or three independent solid cultures grown for 5 days on GMM agar (solid GMM). Densitometry data of TLC plates including standard deviations are represented in the bar graph. WT production levels were assigned a value of 100%, and all other production levels are presented relative to WT. Error bars represent ± one standard deviation.

Similarly to what has been observed in the *hepA* deletion strain, inactivation of *clrD* does not show any evident morphological or physiological phenotype and the mutant grows with the same rate as the isogenic wild type on solid and in liquid media (Figs S1 and S2). Also similarly to *hepA*Δ, the *clrD* inactivated strain displayed higher expression of the ST cluster genes *aflR* and *stcO* under conditions of SM (Fig. 4A) which translates to a slightly increased ST production in liquid GMM (Fig. 4B). The expression pattern of the ST cluster-neighbouring genes AN7826.3 and AN7801.3 remains unchanged in the *clrD*Δ strain, identically to what is seen in *hepA*Δ. Somewhat differently to the *laeA*Δ*hepA*Δ strain, which did show some remediation of *aflR* expression, neither *aflR* nor *stcO* transcripts are detectable in the *laeA*Δ*clrD*Δ background. Consistently, we observed lack of ST production in liquid GMM, but also for this double mutant, a clear remediation of SM production was observed after 5 days' incubation on solid GMM for ST (Fig. 4B) and NOR (Fig. S5).

**Fig. 5:**
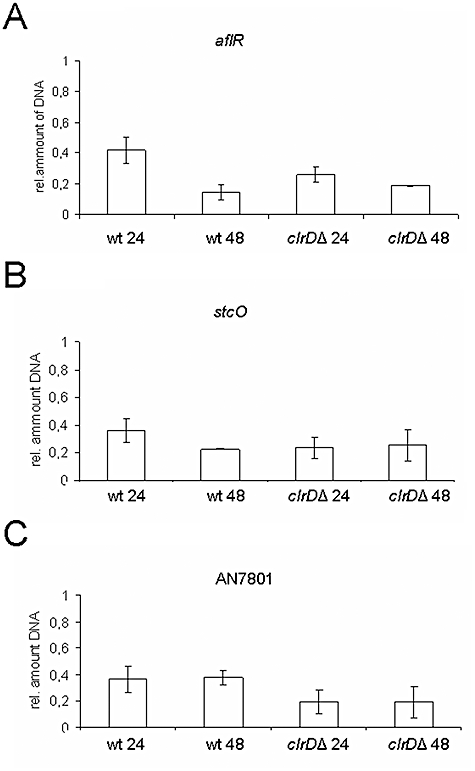
Relative H3K9me3 levels at different gene promoters and physiological conditions. Wild-type (WT) cells cultured in GMM for 24 h (active growth, primary metabolism) or 48 h (stationary phase, SM) were subjected to ChIP analysis probing for trimethylation of H3K9 relative to the total amount of H3 present. The gene promoters of two sterigmatocystin cluster genes (*aflR* and *stcO,* panels A and B, respectively), and the promoter of the telomere-proximal gene AN7801.3 (panel C) were analysed by quantitative PCR. In each experiment, the *clrD*Δ strain was analysed in parallel to determine the non-specific cross-reaction level of the H3K9me3 antibody used in the ChIP reactions. Error bars indicate the standard deviation of two biological and two technical repetitions.

**Fig. 6:**
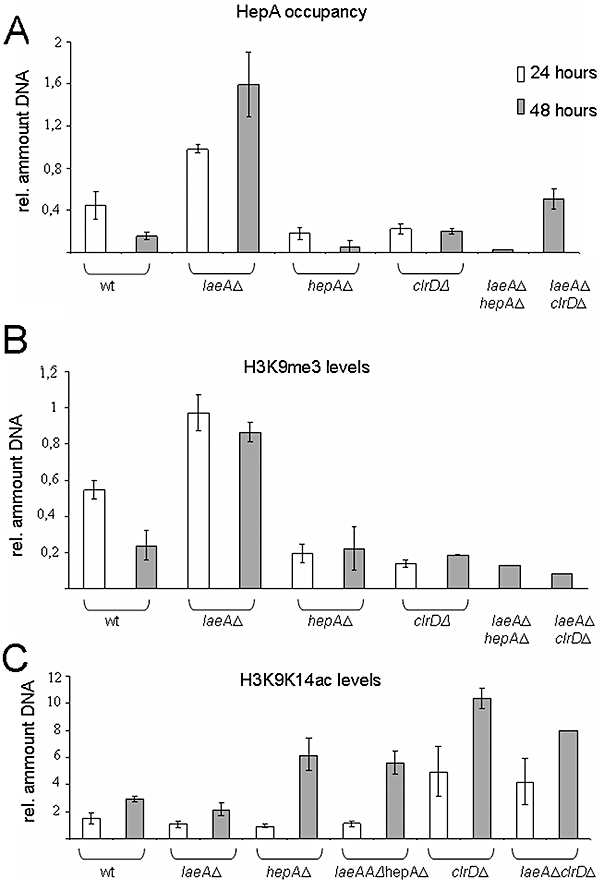
HepA occupancy and the balance between histone H3 methylation and acetylation depends on LaeA. Occupancy by HepA (panel A), relative H3K9me3 levels (panel B) and H3K9/K14 acetylation levels (panel C) were analysed at the *aflR* gene promoter in the relevant strains under different physiological conditions. Wild type (WT), *laeA*Δ, *hepA*Δ, *clrD*Δ, *laeA*Δ*clrD*Δ and *laeA*Δ*hepA*Δ cells were cultured in GMM for 24 h (active growth, primary metabolism) or 48 h (stationary phase, SM) and subjected to ChIP analysis. In each case, quantitative PCR amplified the *aflR* promoter region positioned within the ST cluster. Strains *hepA*Δ (panel A) and *clrD*Δ (panel B) were used as ChIP controls to determine non-specific cross-reaction levels. Error bars indicate the standard deviation of two biological and two technical repetitions.

The slight difference in *aflR* expression between the *laeA*Δ*hepA*Δ and the *laeA*Δ*clrD*Δ strains may indicate that HepA has additional, H3K9me3-independent, functions in ST cluster silencing. However, data from ChIP analysis support a direct involvement of ClrD and histone H3K9me3 levels in the regulation of the ST cluster region. As shown in Fig. 5A and B, trimethylation of H3K9 decreases in the wild type at the *aflR* and *stcO* promoters concomitantly with transcriptional activation (compare Fig. 4A) and depletion of HepA (compare Fig. 3A). This decrease in H3K9me3 is restricted to ST cluster genes and not seen at the AN7801.3 locus (Fig. 5C). Importantly, and consistent with the predicted role of H3K9me for HepA binding, HepA levels are reduced approximately to the *hepA*Δ background in the *clrD*Δ strain (see Fig. 6A).

### The balance between heterochromatic and euchromatic marks is drastically altered in *laeA*Δ

A straightforward interpretation of the results presented above is that LaeA counteracts the heterochromatization mediated by the HepA/ClrD complex. We thus tested the effect of *laeA*Δ on HepA occupancy and H3K9me3 levels at the *aflR* promoter. Figure 6A shows that HepA occupancy at the a*flR* promoter is drastically increased in a *laeA*Δ background both after 24 and 48 h culturing. This increased occupancy correlates strongly with increased H3K9me3 levels (Fig. 6B). Whereas in *laeA*Δ both H3K9 methylation levels and HepA occupancy are greatly increased, this is not the case in the *clrD*Δ*laeA*Δ double mutant, supporting our previous observation that efficient HepA binding requires H3K9me3. However, H3K9me3 levels are also low in *hepA*Δ strains and this suggests that HepA is required for efficient targeting of ClrD to the *aflR* promoter. As expected, the absence of the ClrD methylase (in *clrDΔlaeA*^+^) leads to low HepA occupancy, but HepA is present at wild type levels when both LaeA and ClrD are not functioning (double *clrD*Δ*laeA*Δ). This observation suggests that at the *aflR* promoter HepA shows also a H3K9me3-independent localization and this might explain why remediation for *aflR* expression is seen in *hepA*Δ*laeA*Δ, but not in *clrD*Δ*laeA*Δ.

Taken together, these results suggest a role of LaeA in counteracting H3K9 trimethylation, thus preventing HepA binding and the formation of repressive chromatin. To test whether LaeA is also involved in histone acetylation, an activating euchromatic mark, we analysed by ChIP the acetylation status of histone H3 at lysines K9 and K14 (Fig. 6C). H3K9/K14 acetylation at the *aflR* promoter is higher at conditions of ST cluster activation (48 h culture). H3K9/K14 acetylation is strongly increased in both the *hepA*Δ and in *clrD*Δ strains, regardless whether LaeA is functional or not. These experiments demonstrate that LaeA does not directly participate in the establishment of the activating H3K9K14 acetylation chromatin marks. Moreover, while these crucial marks are correlated with transcriptional activation in the wild type and in the heterochromatin single mutants as well as in the *laeA*Δ*hepA*Δ double mutant, we see strongly increased acetylation also under conditions which do not promote *aflR* expression, i.e. in 24 h cultures of *clrD*Δ and in 48 h cultures of *laeA*Δ*clrD*Δ. Thus, H3K9K14 acetylation levels are not necessarily associated with transcriptional activation of *aflR* but are mainly determined by the methylation level of H3K9. From this set of experiments we can conclude that the balance between acetylated and methylated lysine 9 of histone H3 is determined by HepA and ClrD and that LaeA directly counteracts HepA/ClrD function.

## Discussion

Pre-genomic studies and fungal genome sequencing have identified hundreds of clustered genes known or predicted to be involved in production of polyketides, non-ribosomal peptides or alkaloids (Hoffmeister and Keller, 2007). The contiguous arrangement of genes involved in the synthesis of a given compound might facilitate coordinated gene expression and chromatin domain modifiers thus may regulate such large genomic regions by establishing or lifting chromatin-based gene silencing.

The genetic and biochemical evidence presented here for the first time demonstrates that the establishment of heterochromatin by histone H3K9 trimethylation and subsequent binding of heterochromatin protein 1 (HepA) represents an important regulatory mechanism for secondary metabolite gene expression in *A. nidulans*. Entry of the culture into the stationary growth phase, known to activate SM, generates a signal which leads to upregulation of the general SM regulatory gene LaeA and this protein is subsequently required to turn on transcription of the pathway-specific AflR activator (Bok and Keller, 2004; Bok *et al.*, 2006a). The activation of AflR is a crucial step for the regulation of the ST cluster as it was shown previously that forced ectopic expression of this regulator rendered the ST cluster independent of LaeA. Our data demonstrate that LaeA functions at two levels in *aflR* gene activation. First, LaeA counteracts the establishment of heterochromatin domains at the *aflR* promoter and possibly also in other genes in the ST cluster (shown here only for *stcO* and *stcU*). Second, LaeA also is required for the transcriptional activation step of *aflR* because the absence of heterochromatin marks in strains carrying a *hepA* or a *clrD* deletion is not sufficient to turn on *aflR* expression under conditions of primary metabolism (24 h cultures) where *laeA* is expressed at low levels (compare Fig. S3). Transcriptional activation is often associated with the placement of acetylation marks on chromatin and transcription factors are known to recruit histone acetylation complexes (Li *et al.*, 2007). From our studies it is unlikely that the activating function of LaeA is associated with the H3K9K14 acetylation pathway because these marks are only marginally reduced in the *laeA*Δ strain and even strongly elevated in strains carrying *laeA* deletions in combination with *hepA*Δ or *clrD*Δ. However, we only have tested a very limited set of activating acetylation marks on histones in this study and a broader analysis of activating histones marks, such as other acetylation marks in histones H3 and H4 or methylation of H3K4, will be required to further characterize the pathway through which activation by LaeA operates.

It is worth noting that both heterochromatic marks, H3K9me3 and HepA occupancy, are strongly elevated already under conditions of primary metabolism in the *laeA*Δ strain. This indicates that LaeA function is not restricted to the SM phase but generally functions in balancing heterochromatin and euchromatin establishment in the ST cluster. This raises the possibility that specific de-methylases, which must operate to reduce the H3K9me3 level under conditions of SM in the wild type, do not function in the *laeA*Δ strain. It remains an open question how LaeA prevents hyper-methylation. It could function by stimulating histone H3K9 de-methylases, or by inhibiting the ClrD H3K9 methyltransferase, or both. The presence of a necessary SAM-binding motif in LaeA is no doubt suggestive, but biochemical work will be necessary to mechanistically understand LaeA function and which is the causal link with H3K9 de-methylation.

In conclusion, reversal of heterochromatic marks mediated by LaeA during the onset of SM is required, but is not sufficient, to transcriptionally activate *aflR*. Interestingly, in cultures grown for 5 days on solid GMM expression of genes within the ST cluster become largely independent from LaeA because ST or NOR levels are only reduced for about 50% in the *laeA*Δ strains, depending on the genetic background (Figs 2B and 4B and Fig. S5). These results suggest that prolonged growth on solid medium creates an activation signal for another, LaeA-independent pathway. Because under these conditions we see full restoration and even overproduction of ST and NOR in a *laeA*Δ background by deletion of either *hepA* or *clrD*, it is intuitive to propose that also this alternative, LaeA-independent, pathway employs reversal of heterochromatin during the activation process. However, ChIP analysis of 5 day grown liquid batch cultures was impossible due to DNA degradation (data not shown) and it was not within the scope of this study to establish ChIP conditions for cultures grown on solid medium to be able to test this hypothesis.

H3K9me3 marks and HP1 binding have both been associated with the formation of pericentric heterochromatin and gene repression at euchromatic regions. Available data argue that HP1 can trigger repressive chromatin structure once it is targeted to specific promoters in euchromatin and that the resulting chromatin structure resembles heterochromatin (Hediger and Gasser, 2006). The genes located outside of the ST cluster (e.g. gene AN7826.3 and AN7801.3) are not subject to de-repression in the *hepA*Δ and *clrD*Δ mutants nor are their repressive chromatin marks (H3K9me3 and HepA occupancy) reversed during ST cluster activation. We noticed that the expression level of these genes is sufficiently high to be detected under the conditions of our experimental set-up and thus the high constitutive levels of H3K9me3 and HepA bound to the promoters of these genes do not prevent gene expression. At the moment we have no information on the function of the putatively encoded proteins. However, the specific transcription and chromatin mark profiles argue for a boundary which insulates the ST, and by implication, other gene clusters, from the neighbouring chromosomal domains. Subtelomeric repeat regions present both telomere distal and proximal to the ST cluster might fulfil this insulation function and may in turn, be constitutively heterochromatic.

Chromatin immunoprecipitation analysis of *hepA* deletion strains showed decreased levels of H3K9 trimethylation, but neither *N. crassa hpo* mutants (Freitag *et al.*, 2004) nor *S. pombe* Swi6 mutants (Nakayama *et al.*, 2001) show diminished H3K9me levels. However, HP1 protein interaction partners were shown to include *Drosophila* H3K9 methyltransferases (Aagaard *et al.*, 1999; Yamamoto and Sonoda, 2003) and fly as well as mammalian histone deacetylases (Czermin *et al.*, 2001; Vaute *et al.*, 2002). Our findings could therefore be explained if stable maintenance and spreading of repressive chromatin at the ST locus required interaction between HepA and ClrD and only in the presence of HepA recruitment of more H3K9 methylase activity through a putative HepA/ClrD/HDAC interaction would occur. We find rather surprising that neither deletion of *hepA* or *clrD* show any obvious phenotypic effects. This is in contrast to the crucial functions these genes play in other organisms in maintaining chromosome configuration and stability and the integrity of centromeres and telomeres (Allshire *et al.*, 1995; Ekwall *et al.*, 1995; Kellum and Alberts, 1995; Fanti *et al.*, 1998; Perrini *et al.*, 2004). In *N. crassa* and in *S. pombe*, loss of the single HP1 orthologues led to pronounced growth defects and chromosome loss respectively (Allshire *et al.*, 1995; Freitag *et al.*, 2004). For *Aspergillus fumigatus*, Palmer and coworkers reported that disruption of Af*clrD* results in several growth abnormalities, including reduction in radial growth and conidial production (Palmer *et al.*, 2008). In *A. nidulans*, no reduction in specific growth rates of the *hepA* and *clrD* mutants is seen and hence the effect on secondary metabolite gene expression cannot be attributed to general stress symptoms, which have been implicated in triggering secondary metabolite gene expression in various fungi (Yu and Keller, 2005). Because the majority of the filamentous ascomycetes are predicted to contain single HepA/ClrD orthologues, it is tempting to speculate that coordinated silencing secondary metabolite gene clusters during the active growth phase and reversal of the heterochromatic marks by the conserved LaeA proteins is a widespread epigenetic phenomenon in fungi.

## Experimental procedures

### Fungal strains and growth conditions

Table S1 lists all fungal strains used in this study. *A. nidulans* strains were grown and manipulated according Pontecorvo *et al.* (1953). Some strains are not discussed in the text but were used for sexual crosses to obtain the strains of interest. All strains were maintained as glycerol stocks and were grown at 37°C on glucose minimal medium with appropriate supplements (GMM). Transformations were performed as described previously (Tilburn *et al.*, 1983).

### Construction of deletion/fusion cassettes and strains

All cassettes were constructed by DJ-PCR (Yu *et al.*, 2004) and Table S2 lists all oligonucleotides used for this purpose. Deletion of the *hepA* and *clrD* open reading frames were carried out by replacing each of them with the *A. fumigatus pyrG* gene (*pyrGf*) and details of knock-out construct generation as well as generation of *hepA* and *clrD* complemented strains are given in *Supporting information*.

### Nucleic acid analysis

Standard techniques were used to analyse and manipulate RNA and DNA. To characterize deletion strains and complementation events in these strains, we used analytical PCR and Southern blotting. Restriction enzyme digests and probes used for Southern analysis, RNA extraction protocols and probes for Northern analysis are described in detail in *Supporting information*. Growth conditions for expression analysis are described in the legends to figures.

### Secondary metabolites analysis

Thin-layer chromatography (TLC) analysis was applied for ST and NOR production of *A. nidulans* wild-type and mutant strains from either 24 or 48 h liquid shake cultures on GMM or on GMM solid media cultures using published procedures (Bok and Keller, 2004). Details for the SM analysis carried out during this work are given in *Supporting information*.

### Statistical analysis

Standard deviation was calculated from at least three independent experiments. In TLC analysis of the wild type, where the level was arbitrarily set to 100%, the relative SD_rel_ was calculated proportionally to the absolute densitometry value *aV*, SD_rel_ = [(100/*aV*) × SD*_aV_*]. For statistical analyses of metabolite production, a probability of type I error of less than 0.01 was considered statistically significant. Where only two treatments were compared, significance of variation was determined using a Student's *t*-test. Where more than two treatments were compared, analysis of variance was used to determine significance of overall variability among treatments, followed by a Neumann–Keuls test to compare individual pairs of treatments. The Microsoft Excel data analysis package was used to perform analysis of variance and *t*-tests. Neumann–Keuls tests were performed by hand.

### Chromatin immunoprecipitation coupled to quantitative PCR analysis

Chromatin immunoprecipitation was carried out as described previously (Bernreiter *et al.*, 2007). Antibodies used for ChIP were purchased from Abcam, Cambridge, UK (rabbit polyclonal to histone H3 trimethyl K9, ab 8898, rabbit polyclonal to HP1, ab24726 and rabbit polyclonal to human C-terminus histone H3 antibody, ab1791) and from Upstate-Millipore, MA, USA, Anti-acetyl K9-K14 histone H3, 06-599. Two micrograms of antibody was used per reaction of 200 µg total protein. To detect the background signal of the immunoprecipitation reaction while using anti-HP1 antibody a *hepA*-deleted strain was analysed in parallel.

Amplification and detection of precipitated DNA in real-time qPCR was performed with Platinum SYBR Green qPCR SuperMix UDG (Invitrogen) following the instructions of the provider. The relative amounts of DNA were calculated by dividing the immunoprecipitated DNA by the input DNA. Each PCR reaction was replicated. To normalize the amount of DNA precipitated with histone H3-trimethyl K9 and H3-acetyl, the resulting ratio of the precipitation with these antibodies was divided by the resulting ratio of the anti-C-terminus histone H3 precipitation. Two biological repeats were performed for each condition and standard deviation was calculated upon these. Primers aflRpromfwd and aflRpromrev were used to amplify *aflR* promoter region, stcOpF and stcOpR were used to amplify *stcO* promoter region, AN7801pF and AN7801pR were used to amplify AN7801 promoter region, niiA_nuc-1_F and niiA_nuc-1_R were used to amplify *niiA* nucleosome 1 region.
