# Supplementary material for: Heterochromatic marks are associated with the repression of secondary metabolism clusters in Aspergillus nidulans
Source: Mol Microbiol. 2010 Feb 1;76(6):1376–86. doi: 10.1111/j.1365-2958.2010.07051.x (PMC2904488; doi:10.1111/j.1365-2958.2010.07051.x)
Supplement: Supplementary file 1 [file mmi0076-1376-SD1.pdf]

Reyes-Dominguez et al: Supporting Information (Experimental procedures, Tables and Figures S1 to S8)

Experimental procedures

Nucleic acid analysis details for probes and restriction digests.

To identify the strains deleted for *hepA*, a Southern blot with genomic DNA digested with *SacI* was hybridized with a ^{32}P labeled probe of 3.4 Kb PCR product amplified by HP1840F and HP5270R. A single 5 Kb band confirmed *hepA* deletion and single genomic integration of the deletion cassette (not shown). The same membrane was stripped and hybridized with a ^{32}P labeled probe of 389 bp PCR product amplified by HP5530F and HP5290 corresponding to *hepA* ORF. No signal was detected for the *hepA* deleted strain. To identify the *clrDΔ* strains, a southern blot with genomic DNA digested with *Kpn*I was hybridized with ^{32}P labeled probe of 3.8 Kb PCR product amplified with primers clrD161F and clrD3519R. A single 8.5 Kb band confirmed *clrD* deletion and single integration of the deletion cassette. The same membrane was stripped and hybridized with a ^{32}P labeled probe of 812 bp PCR product amplified by clrDAspF and clrDAspR corresponding to *clrD* ORF. No signal was detected for the *clrD* deleted strain (not shown). In order to identify the complemented *hepA* transformants a southern blot with genomic DNA digested with *Xba*I was hybridized with ^{32}P labelled probe of 756 bp PCR product amplified by ArgflaF and ArgflaR primers. A 11 Kb band confirmed single *hepA* integration in *argB* locus (not shown). In order to identify the integration of *clrD* gene in a *clrDΔ* background, a southern blot with the transformants DNA was performed. Genomic DNA was digested with *BamH*I, blotted and hybridized with ^{32}P labelled probe of 756 bp PCR product amplified by ArgflaF and ArgflaR primers. A 7 Kb band confirmed single *clrD* integration in *argB* locus (not shown). Total RNA was extracted from *Aspergillus* strains using Trizol reagent (Invitrogen Co.) according to the manufacturer's instructions. RNA blots were hybridized with ^{32}P labeled probes made from a 1.3 Kb *EcoR*V-*Xho*I fragment from pJW19 containing the *aflR* coding region, a 3 Kb *Hind*III fragment from pJW45-4 containing the *laeA* coding region, a 1.1 Kb *Eco*RI-*Hin*dIII fragment from pUCHH(458) containing the *ipnA* coding region, or from PCR products (amplified from *A. nidulans* wild type DNA) from the following regions: a 1 Kb

tdiB[12] PCR product (amplified by primers NAIf1 and NAIr1), a 859 bp *hepA* PCR product (amplified by HpFwd and HpRev), a 864 bp *stcO* PCR product (amplified by stcOF and stcOR), a 991 bp AN7826.3 PCR product (amplified by corAF and corAR), a 2917 bp AN7801.3 PCR product (amplified by 7801F and 7801R), a 478 bp actin PCR product (amplified by ActFW and ActRV) and a 1 Kb *clrD* PCR product (amplified by crlDFWD and crlDREV). For Reverse transcription quantitative real-time PCR, cDNA was synthesized using the SuperScript™ First-Strand Synthesis System (Invitrogen) following the instructions of the provider. The platinum SYBR green qPCR SuperMix-UDG (Invitrogen) was used for amplification and detection of DNA in qPCR using a thousand fold dilution of the cDNA. *aflR* transcription was assessed using the primers aflRorfF and aflRorfR, *hepA* with primers HP3F and HP3R and *clrD* with primers clrDorf 2 F and clrDorf 2 R. All signals were normalized to the constitutively transcribed actin gene (*acnA*) amplified with primers acnf and acnR. The Bio-Rad (Hercules, CA) MyiQ cycler was used as device.

Construction of deletion/fusion cassettes and strains.

A deletion cassette for *hepA* was constructed were *pyrGf* was flanked by 3' and 5' sequences of the *hepA* ORF. The 5'-flanking *hepA* sequence was amplified from *A. nidulans* wild type genomic DNA using primers HP1840F and HP5270R, and the 3'-flanking sequence was amplified from *A. nidulans* wild type genomic DNA with HP6090F and HP9600R. *pyrGf* was amplified from *A. fumigatus* wild type genomic DNA with primers pyrGHPF and pyrGhHPR. Nested primers HP2240 and HP9029 were used to amplify the complete assembled molecule. The *hepA* deletion cassette was used to transform a *pyrG89 pantoB100 riboB2 argB2 yA2* strain. Transformants were selected on minimal media with appropriate supplements omitting uracil and uridine.

The deletion of *clrD* ORF was carried out by replacing it by the *pyrGf* gene (*pyrGf*). A deletion cassette was constructed were *pyrGf* was flanked by 3' and 5' sequences of the *clrD* ORF. The 5′-flanking *clrD* sequence was amplified from *A. nidulans* wild type genomic DNA with the primers *clrD*161F and *clrD*3113R and the 3'-flanking sequence was amplified with primers clrD4758F and clrD7120R. pyrGf was amplified from *A. fumigatus* wild type genomic DNA with primers clrDpyrF and clrDpyrR. Nested primers clrDNestF and clrDNestR were used to amplify the complete assembled molecule. The

clrD deletion cassette was used to transform a *pyrG*89 *pyroA*4 *argB*2 *yA*2 strain. Transformants were selected on minimal media with appropriate supplements omitting uracil and uridine.

Complementation of *hepA* in a *hepAΔ*strain.

In order to introduce *hepA* in a *hepAΔ::pyrG89*, *pantoB100*, *riboB2*, *argB2* strain, a fusion *hepA-argB* molecule was constructed. *hepA* was amplified from *A. nidulans* wild type genomic DNA with primers HpmF and HpmR and fused with the 2.16 Kb PCR product amplified from plasmid pFB39 (carrying the *argB* gene) with primers HpmArgF and argBdownR. The assembled molecule was amplified with primers hpmNesF and argBbglIIR and used to transform *hepAΔ::pyrG89*, *argB2*, *pantoB100*, *riboB2*. Transformants were selected on minimal media with appropriate supplements in the absence of arginine.

HepA Western, plasmid and strain constructions for over-expression of S-tagged HepA

In order to over-express *hepA*, a transcriptional fusion between the S-tagged *hepA* coding sequence and the *alcA* promoter was generated. 952 bp of the HepA coding sequence was amplified with HPF and HPR primers, in which the HPF primer contains additional bases coding the for the S-tag and a *BamH*I site. The HPR primer contains a *Kpn*I restriction site. The amplified fragment was introduced into *Kpn*I-*BamH*I sites of pMT-mRFP (Toews *et al.* 2004), which contains the *alcA* promoter and *argB*. The terminator of *trpC* was amplified from plasmid phER-trpC (Pachlinger et al, 2005) as 590 bp *KpnI* fragment and introduced into the plasmid at the 3′ end of the HepA coding region with a *Kpn*I restriction site. The resulting plasmid was sequenced to verify the correctness of the HepA-ORF and used to transform YR257 Δ*hepA*: *pyrG argB2 pantoB100 riboB2 yA2 veA1*. Transformants were selected on minimal media with appropriate supplements without arginine and confirmed by PCR and Southern hybridization (not shown). For Southern analysis genomic DNA was digested with *BamH*I and hybridized with a ^{32}P labelled probe derived from a 520 bp PCR product amplified by HP5580F and Kpn1-5′R. A single 6 KB band confirmed a single genomic integration of the HepA overexpression construct and one of the correct strains was designated *alcAp*-**S**-*hepA* and further used in the Westerns.

Growth and conditions: *Aspergillus nidulans* strains wild type (*hepA*$^{+}$), *hepA*Δ, and *alcAp-S-hepA* were grown in 250ml Erlenmeyer flasks containing 50ml 0,1 % fructose minimal medium and 10 mM sodium nitrite. Cultures were grown at 37°C with shaking (180 rpm) for 16 hours. To overexpress *hepA* from the *alcA* promoter, cultures were induced for 2 hours with 50 mM EMK (ethylmethylketone). For non-induced conditions the samples were grown without the inducer and both induced and non-induced cultures were collected at same time. Mycelia were harvested by filtering and frozen in liquid nitrogen.

<u>Western blot:</u> Proteins were extracted using a modified TCA protein extraction protocol. Mycelia were homogenized in liquid nitrogen to a fine powder. 0.1 grams of powder was suspended in 1ml of TCA buffer (10mM Tris pH 8, 20mM KCl, 1mM EDTA and 12% TCA), vortexed vigorously and centrifuged for 15 minutes at 13.000 rpm at 4°C and the supernatant was discarded. The pellet was resuspended and washed in 1ml Tris Base (1M pH unadjusted) to remove remaining TCA from the pellet and the supernatant discarded. The pellet was resuspended in 200 µl of sample buffer (0, 3% SDS, 0,1M Tris.HCl pH 7,0) , heated to 95°C for 5 minutes and centrifuged for 15 minutes at 13000 rpm and 4°C to remove cell debris. Protein concentration in the supernatant was quantified using the BCA protein quantification kit (Pierce, USA). Roughly 20 µg of proteins were used for SDS-PAGE and Western blotting, and the membrane was probed with HP1 (1:200) antibody and S-tag antibody from Abcam (#19321) in a 1:10.000 dilution.

Complementation of *clrD* in a *clrDΔ* strain.

In order to introduce *clrD* in a *clrDΔ::pyrG89*, *pyroA4*, *argB2*, *yA2* strain, a fusion *clrD-argB* molecule was constructed. *clrD* was amplified from *A. nidulans* wild type genomic DNA with primers clrDMinF and clrDMinR and fused with the 2.16 Kb PCR product amplified from pFB39 plasmid (carrying the *argB* gene) with primers dimMRargBF and argBdownR. The assembled molecule was amplified with primers clrDmNestF and argBbglIIR and used to transform *clrDΔ::pyrG89*, *argB2*, *pyroA4*, *yA2*. Transformants were selected on minimal media with the appropriate supplement in the absence of arginine.

Western blot: Mycelia (18 h of growth) were collected from wild type and *clrD*Δ strains, washed with minimal media, dried and grounded in liquid nitrogen. The powder was weighed and suspended in 2 ml cold buffer A (50 mM Tris, pH 7.5, 5 mM $MgOAc_2$, 20% glycerol, 5mM EGTA, 3 mM $CaCl_2$ 100, 1M Sorbitol, 7% Ficoll, 5 mM DTT, Protease inhibitors cocktail Sigma, 1:500) per gram. Once homogenised, 4 ml of buffer B (25 mM Tris, pH 7.5, 5 mM $MgOAc_2$, 10% glycerol, 5mM EGTA, 5 mM DTT, Protease inhibitors cocktail Sigma, 1:500) per gram were added. This suspension was centrifuged at 1500g for 7 min at 4 °C. Whole cell extracts were mixed with SDS sample buffer (10% glycerol, 100 mM DTT, 2% SDS, 0.1% bromophenol blue), incubated at 95°C for 4 min and analysed by electrophoresis on 15% polyacrylamide gel. They were transferred to nitrocellulose membrane and probed either with polyclonal antibody against the C-tail of histone H3 (1:1000) or with polyclonal to histone H3 tri methyl K9 (1:1000).

Secondary metabolites analysis.

For solid media, a cork borer with diameter of 1.2 cm was used to collect samples from each plate after 72 hr growth. Each sample was homogenized and mixed well with 3 ml of double distilled H_2O. Then 3 ml of $CHCl_3$ was added and agitated with a vortex. After centrifugation for 5 min at 1,000 rpm, the separated organic phase was transferred to an 15 ml conical tube. All samples were dried down and resuspended in 100 µl $CHCl_3$. Twenty-five µl of each extract was spotted on a TLC plate (Whatman Ltd, 0.25 mm silica gel 60 F254 and fluorescent indicator) and separated in toluene: ethyl acetate: formic acid (40:60:0,5). The TLC plates were sprayed with aluminium chloride to enhance ST fluorescence upon exposure to long wave (365 nm) UV light.

For liquid shake cultures, *A. nidulans* strains were inoculated from conidia suspensions into 50 ml of liquid GMM in 125 ml flasks to a final concentration of 10^6 conidia/ml. Flasks were then incubated at 37°C for 48 or 72 hours with shaking at 250 rpm. 25 ml acetone was added to liquid culture, mixed well with a vortex and filtrated to remove mycelia. Acetone was evaporated over night in fume hood. The next day 50 ml chloroform was added to the filtrate, mixed well and then centrifuged for 10 min at 2,500 rpm. Chloroform layers (40 ml) were transferred to new tubes, dried and 100 µl chloroform was added to resuspend extracts in each tube. Thirty µl of each extract was separated on a TLC plate for ST analysis. Quantification of ST and NOR from *A.*

nidulans extracts was accomplished using a CAMAG II densitometer, according to manufacturer's instructions. A wavelength of 245 nm was used for all analyses.

Quantification of radial growth.

Aspergillus nidulans strains wild type (RJW84.5), *hepAΔ* (RJW63.1) and *clrD*Δ (YR31.1) were grown on solid GMM. Twenty µl of a spore suspension containing 10E+4 spores per ml (in 0.1% aqueous Tween80) were inoculated at the centre of a plate. The diameter of the colony was measured at a time period of every 24 hours.

Quantification of biomass.

Aspergillus nidulans strains wild type (RJW84.5), *hepAΔ* (RJW63.1) and *clrD*Δ (YR31.1) were grown on liquid GMM. 2.5 E+8 conidia were inoculated in 250ml Erlenmeyer flasks containing 50ml GMM. The strains were incubated at 37°C with shaking at 180 rpm for five different time period of analysis i.e. 0, 4, 8, 16, 24, 48 and 72 hours. Mycelia was harvested by filtering, washed with sterile deionised water and squeezed in paper towel to get rid of all the liquid, packed in pre weighted aluminium foil and dried in a 65°C oven for three days. Weight of dry mass was measured for two consecutive days.

Quantification of conidial production.

Aspergillus nidulans strains wild type (RJW84.5), *hepAΔ* (RJW63.1) and *clrD*Δ (YR31.1) were grown on solid GMM media which were inoculated at the centre of the plate with 20µl of a spore suspension containing 10E+4 spores per ml (in 0.1% aqueous Tween80 solution). The conidia from the entire colony were collected after 5 days of incubation in 0.01% Tween20 and counted with a haemocytometer.

Supplementary Table 1. Strains used in this study.

Strains	Genotype	Source
RJW84.5	*wA3, veA1*	This study
RJW61.1	*wA3, laeAΔ::metG, veA1*	This study
RJW63.1	*wA3, hepAΔ::pyrG, veA1*	This study
RJW63.6	*wA3, hepAΔ::pyrG; laeAΔ::metG, veA1*	This study
RJW132.3	*wA3, clrDΔ::pyrG, veA1*	This study
RJW133.4	*wA3, clrDΔ::pyrG, laeAΔ::metG, veA1*	This study
RJW49.1	*laeAΔ::metG, laeA::trpC, veA1*	This study
RJW61.14	*wA3, stcEΔ::argB; veA1*	This study
RJW61.9	*wA3, stcEΔ::argB; laeAΔ::metG, veA1*	This study
RJW63.8	*wA3, stcEΔ::argB; hepAΔ::pyrG, veA1*	This study
RJW63.6	*wA3, stcEΔ::argB; hepAΔ::pyrG; laeAΔ::metG, veA1*	This study
REKS8.9	*wA3, clrDΔ::pyrG; stcEΔ::argB; veA1*	This study
REKS8.3	*wA3, clrDΔ::pyrG; stcEΔ::argB; laeAΔ::metG, veA1*	This study
YR1108	*clrDΔ::pyrG, pyroA4, argB2, yA2, veA1*	This study
YR1208	*clrDΔ::pyrG, laeAΔ::metG, yA2 , veA1*	This study
YR1110	*hepAΔ::pyrG, hepA-argBbgl*II, *pantoB100, riboB2, yA2, veA1*	This study
YR1109	*clrDΔ::pyrG, clrD-argBbgl*II, *pyroA4, yA2, veA1*	This study
YR1208	Δ*clrD::pyrG,* Δ*laeA::metG, yA2, veA1*	This study
YR33.1	Δ*clrD::pyrG, wA2, veA1*	This study
OE::HepA	*alcAp::*Stag-*hepA-argB,* Δ*hepA::pyrG, argB2, pantoB100, riboB2, yA2, veA1*	This study

Supplementary Table 2. Oligonucleotides used in this study.

Oligonucleotide name	Sequence (5´to 3´)
HP1840F	AAGCCTGGATAAATCTCCGTTCGTGC
HP5270R	TGTTCGCGACGAAGTGAAGCTGAACAG
HP6090F	TTTCATACATACCCTCGGAATTCCCG
HP9600R	ACCAGGCCAGGAATCCGCAGTTTCGGC
pyrGHPF	AAGCACCGTCAAACTGTTTTCGCGTCTGTTCAGCTTCACTTCGTCGCGAACA CGCGCAAACAGAATTCGCCTCAAACAATGC
pyrGhHPR	TGGTGTTAGGTTTGCGGGAATTCCGAGGGTATGTATGAAAGAATTC TCAGTCCTGCTCC
HP2240	TTCTTCCGCGCGACCGTTCACAAGAG
HP9029	AGGATAGCGCTTCATCTCCTCCGCG
clrD161F	GGTTCAGCGGACCGCCTGATAACCACCT
clrD3113R	GTCAGTTGCCATAAGCTTGATAGTG
clrD4758F	GATATTACCTATGCCTTTGTCT
clrD7120R	GCAAATGACATCTCCCATCATGTC
clrDpyrF	TATTTACTTTTTGGCGTCCCACTATCAAGCTTATGGCAACTGACAGGGGTG AATTCGCCTCAAACAATGC
clrDpyrR	AGAGACAGAAGAGAAATCTACCTTGCTTTCTTCCTCTCAGTAGCCCACAGG AATTCTCAGTCCTGCTCC
clrDNestF	CGTGTTTGATTTCTACAAGC
clrDNestR	AGTCTTACACTCGCTCATTCACTCG
HpmF	AGTACAAGGTAGAATGAGGGGC
HpmR	ACGTGATATTCAGCGGGTCATAGAGAA
HpmArgF	CGCCATGCTTCTCTATGACCCGCTGAATATCACGTATTTCGCGGTTTTTT GGGGTAGTCATCTAATG
argBdownR	GTCGACCTACAGCCATTGCGAAACCTC
hpmNesF	TCCTGGTGAGGAGTTATTATCAGG
argBbglIIR	ATTGGTTTGCAGAAGCTTTCCTGGTTTCG
clrDMinF	CGCCGCCGTCACCGGGCGCCAGATC
clrDMinR	GAATGCAATAGGATAGAACCTCACG
dimMRargBF	ATTGCATTGCGTCGTGAGGTTCTATCCTATTGCATTCAAGCTTTATTTCG CGGTTTTTTG

Supplemen

clrDmNestF	GATACCACAGTGACGAGTGGGGAGC
argBbglIIR	ATTGGTTTGCAGAAGCTTTCCTGGTTTCG
HP1840F	AAGCCTGGATAAATCTCCGTTCGTGC
HP5270R	TGTTCGCGACGAAGTGAAGCTGAACAG
HP5530F	CGACGATGCTGGCTGACGTTGCGAGAAGG
HP5290	ACTTCGGCACCCAATCGGGCGCTACG
clrD161F	GGTTCAGCGGACCGCCTGATAACCACCT
clrD3519R	TGCGCGCCTTTCAAGAGCATCTGT
clrDAspF	TCGAGTTCATCAACGAGTACAAGC
clrDAspR	CTGCCCGCGACAATTTGGCTCG3
ArgflaF	GGGCTATCAAGTCGGGGTCAA
ArgflaR	CATGAACTTCCAGCCCTCCTTAG
HpFwd	TTCTCACTGACACTCTCGCAGC
HpRev	TAAAAAGCGCTGGATGACCCAAG
stcOF	ATGCCTTCGTACGCCCTTCTAG
stcOR	AAGCAGCAACAAAATACAAACCCC
corAF	TGAACTGTCCCTCCAATACTCCC
corAR	AACATTCATACCATACCATCCTGC
ActFW	CAGTCCAAGCGTGGTATCC
ActRV	CATCAGGTAGTCCGTCAGG
crlDFWD	CAGACACTTGCACACCGCACTC
crlDREV	TCCAGTGTGGAAAATCTCCAGGC
aflRorfF	CAGGGTGGTCGACGACAAGGGGGT
aflRorfR	AGCCCAGCTGGTGCTGAGCGAGCTATAC
HP3F	GACGCAGAAGGTCACGAGCA
HP3R	GAGCATTTTGCGTGGGCATT
clrDorf 2 F	GCGTAACGCCCCTTCCTATC
clrDorf 2 R	GGTAGTCATCGCCGTGGGTA
acnf	CGAGCGCGGATACACCTTC
acnR	TACGGACGTCGACATCACAC
aflRpromfwd	GTACAGCAAAGACAAAGATATGCG
aflRpromrev	GTACTGTGTAAGCTGTACTTGGG
stcOpF	CCTGATGTAGGATTAGGATAGAATG
stcOpR	AAGGCTGGGCTGAAGATTATCGAG
AN7801pF	TGTACCCAATAGCGTCCCATCATAAG
AN7801pR	CACACTTCAACTCTGTAATCCAAGG
niiA_nuc-1_F	GGAAATTCAGGCAGTGCATC
niiA_nuc-1_R	TAGGATCTGGAGTGTGGCTAGAG
7801F	AGCATCGCGATGAACGAGCCC3
7801R	AATTGACCAAACAATCGCCAGGG
HPF	GGCGGCGGATCCATGAAGGAGACCGCCGCCGCCAAGTTCGAGCGCCAGCACATG GACTCCCCGCGTGGGTCTATCAATTGGCTTTTTCTG
HPR	GGCGGCGGTACCTTACGCATCCGAGTCTTTAAAAACTAAGTGT
Kpn1-5´R	AAAGGTACCTTC TAGAAAGAAGGATTACC

Reyes et al., Supporting Figure S1

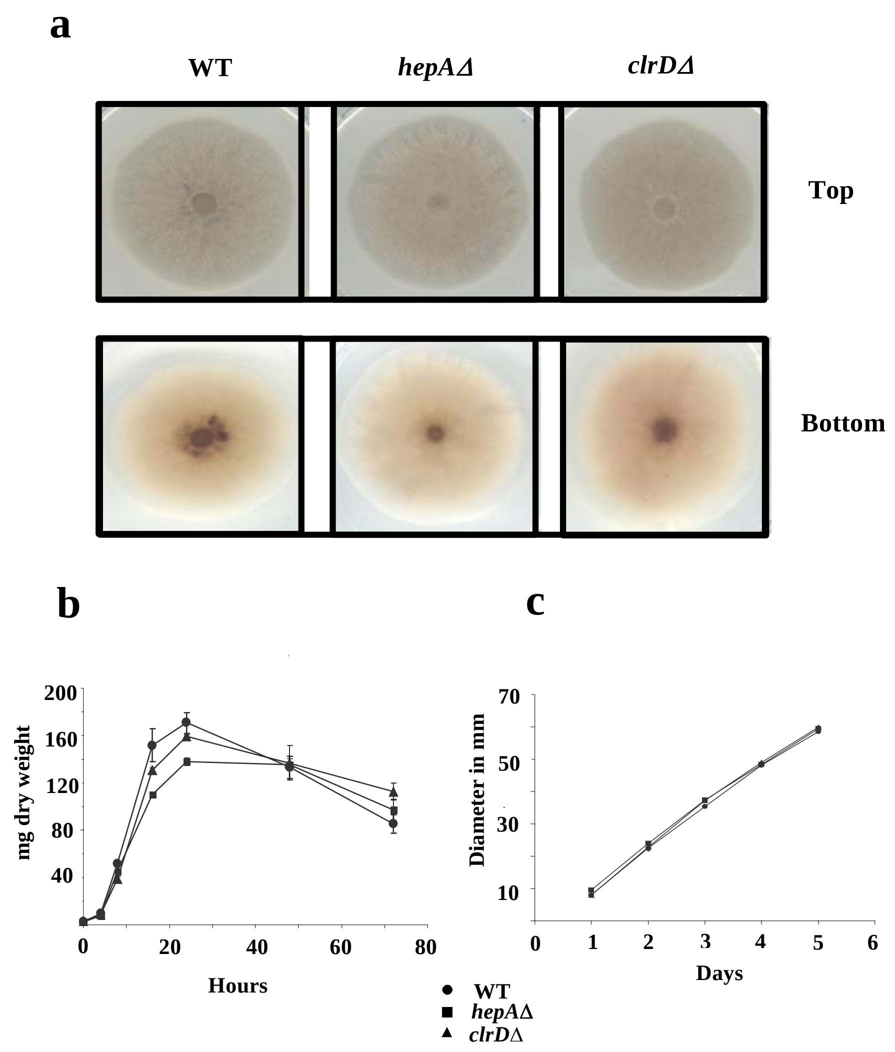

Supporting Figure S1. Growth of wild type (*hepA*$^{+}$ *clrD*$^{+}$), *hepA*Δ and *clrD*Δ strains inoculated and cultured as described in Supplementary Materials and Methods. a) Top and bottom view of relevant strains growing on solid GMM. All strains carry the *wA2* mutation and display a white spore colour (top view). A slightly lighter pigmentation (bottom view) in mutant strains can be noticed as the only phenotypical difference between the wild type, *hepA*Δ, and *clrD*Δ strains. b) Comparison of biomass accumulation of relevant strains. Dry weight was measured after 4, 8, 16, 24, 48 and 72 hours of growth in liquid GMM. Each value represents the mean of three independent experiments. c) Quantification of radial growth of relevant strains. The diameter of the colony was measured at a time interval of 24 hours of growth at 37°C.

Reyes et al., Supporting Figure S2.

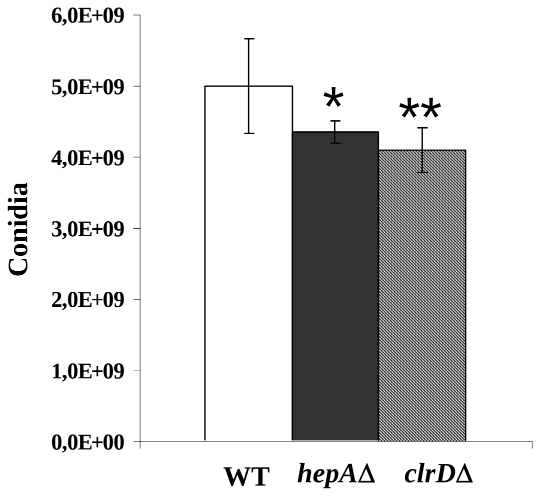

Supporting Figure S2. The deletion of *hepA* or *clrD* does not affect conidiation. *Aspergillus nidulans* strains wild type (*hepA*$^{+}$*clrD*$^{+}$), *hepA*Δ, and *clrD*Δ were inoculated with a defined density (see for details Supplementary Materials and Methods) and grown on solid GMM at 37°C. After five days of incubation, the conidia from the entire colony were collected and counted with a haemocytometer. Error bars indicate standard deviation. (* P = 0.17948, ** P = 0.10454).

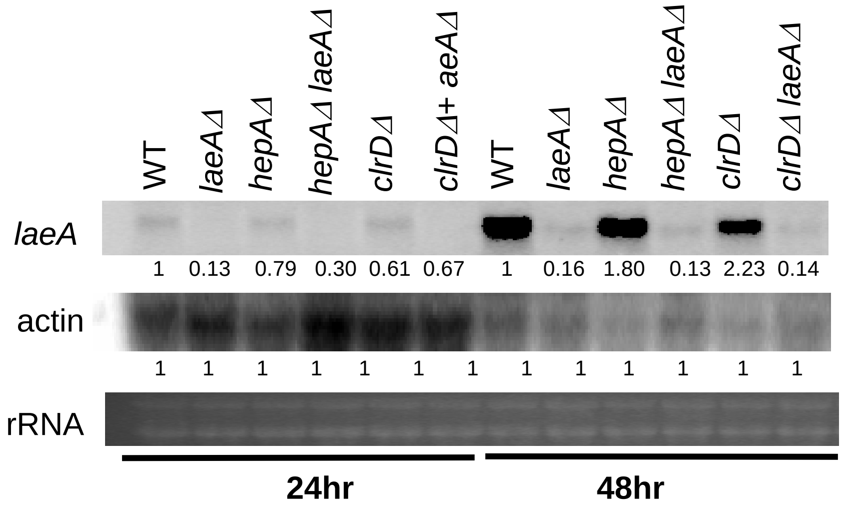

Supporting Figure S3. The expression level of *laeA* is only slightly altered in heterochromatin mutants. Comparison of mRNA steady-state levels of the *laeA* gene in strains grown in liquid GMM for for 24h (primary metabolism) or 48h (secondary metabolism). Strains are *laeA*$^{+}$*hepA*$^{+}$*clrD*$^{+}$ (wild type, WT) or are deleted for l*aeA*, *hepA* or *clrD*,as single genes or in combinations. *laeA* expression is shown along with the actin (*acnA*) and rRNA loading controls. Numbers below each lane are actin-normalized relative expression levels of the mutant strains in relation to the actin-normalized wild type control, for which the expression level at 24 hours has been arbitrarily set to 1.

Reyes et al., Supporting Figure S4.

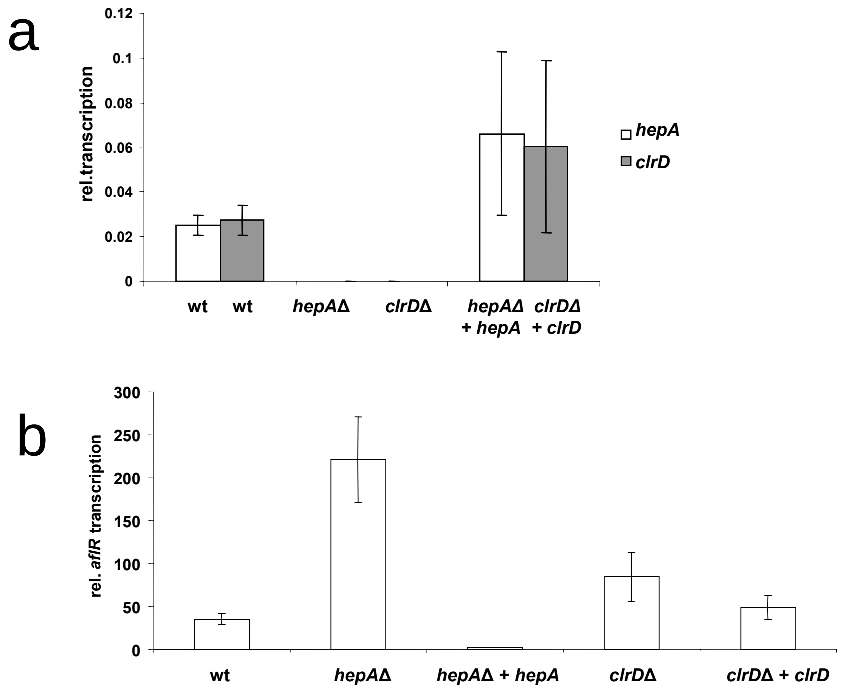

Supporting Figure S4. The phenotypes of the *hepA* and *clrD* deletions are reverted in the complemented strains. **a.** The mRNA levels of *hepA* (open bars) and *clrD* (filled bars) in relation to the actin gene mRNA (see Supplementary Methods) were measured by quantitative RT-PCR (qRT-PCR) after 48 hours of culture in GMM and are shown for the wild type (wt), the deletion strains (*hepA*Δ, *clrD*Δ) and the complemented strains (*hepA*Δ+*hepA*, *clrD*Δ+*clrD*). Specific messengers are absent in the deletion strains (*hepA*Δ and *clrD*Δ). When the deletions are complemented by stably inserting the full gene into the *argB* locus, expression is restored and even increased two to three fold. This position effect of genes inserted into the *argB* locus has been reported previously (Oestreicher *et al.*, 2008) **b**. The re-insertion of *hepA* and *clrD* into the respective deletion strains at the *argB* locus results in a reversal of the deletion phenotypes, i.e. reversal of strong (in *hepA*Δ background) or moderate (in *clrD*Δ background) overexpression of *aflR*. mRNA levels of a*flR* were measured by qRT-PCR in the corresponding strains after 48 hours of culture in GMM.

Reyes et al., Supporting Figure S 5

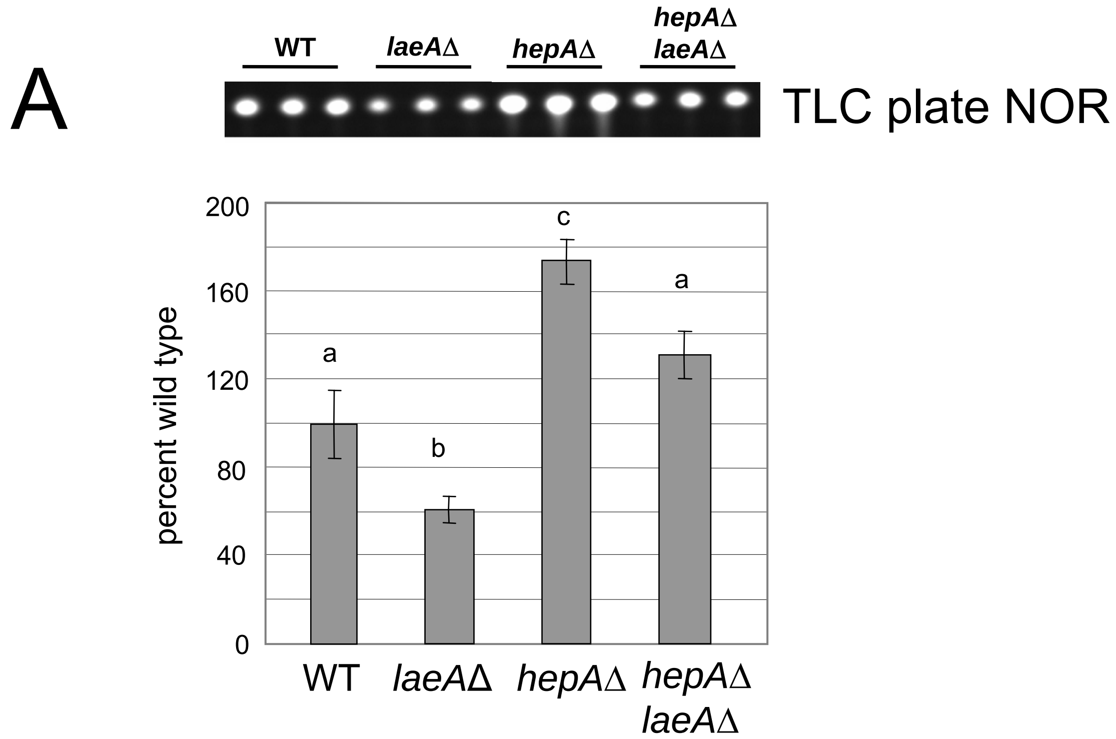

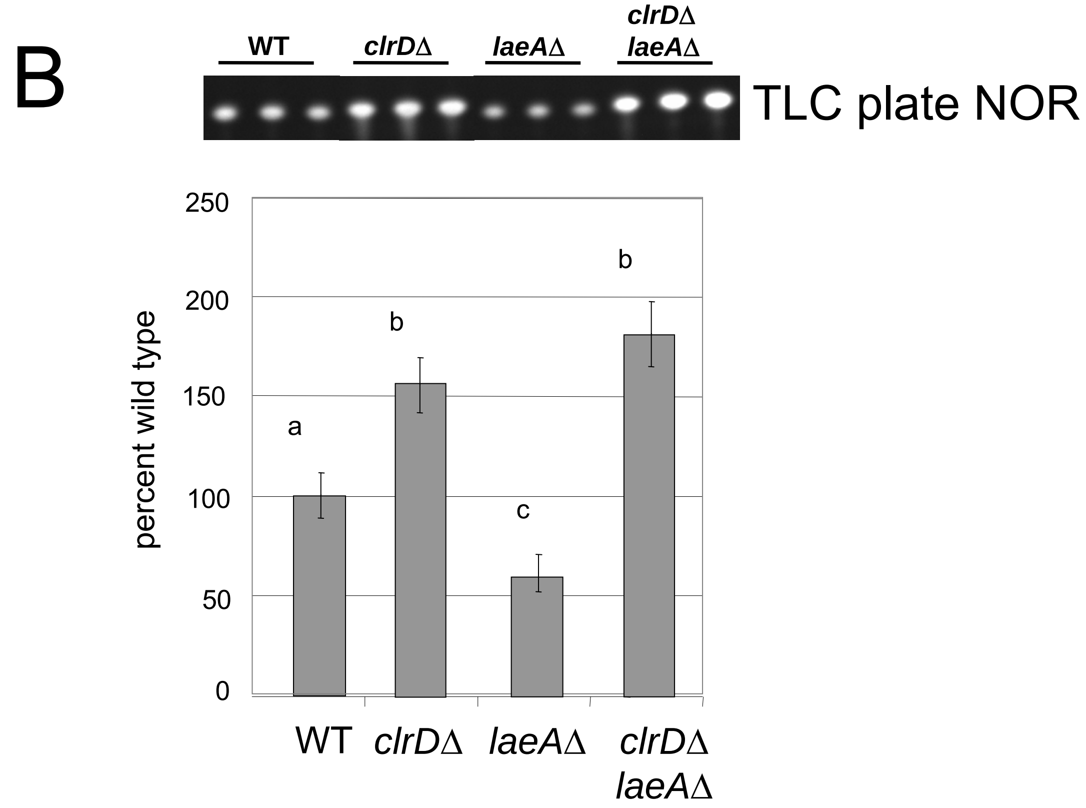

Supp. Figure S5

TLC-plate analysis of secondary metabolites produced on solid GMM. In panel A, NOR production was quantified in a *hepA*Δ strain and compared to wild type, *laeA* Δ and *hepAΔ laeAΔ* strains. In panel B, NOR amount was compared between a *clrD*Δ strain and wild type, *laeA*Δ and *clrD*Δ *laeA*Δ strains Bar-graphs below the TLC plates summarize the results from three independent GMM solid media cultures grown for 5 days, extracted and analyzed separately for their production of norsolorinic acid (NOR). NOR amount was determined by densitometry and subjected to statistical analysis as described in the Experimental Procedures section. WT production levels were assigned a value of 1, and all other production levels are presented relative to WT. Different letters above columns represent statistical differences at p=0.01. Error bars represent +/- one standard deviation.

Reyes et al., Supporting Figure S6

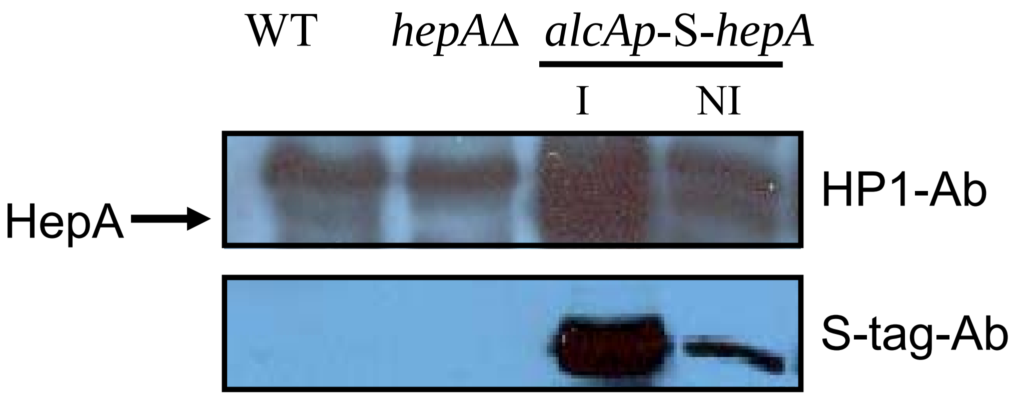

Supporting Figure S6. Western blots of HepA. Wild type (WT), *hepA*Δ and a strain containing a construct for overexpressing S-tagged *hepA* under the *A. nidulans alcA* promoter (*alcAp-S-hepA)* were grown overnight on 0.1% fructose minimal media (NI, non-induced, derepressed conditions for *alcAp). S-hepA* over-expression was induced by addition of 50 mM ethylmethylketone for two hours (I); roughly 20 µg of proteins from a total protein extract were used for SDS-PAGE and Western blot was probed with the HP-1 antibody (HP-1-Ab)used also for ChIP, in a 1:200 dilution or with an antibody recognizing the S-tag (S-tag-Ab).

Reyes et al., Supporting Figure S7

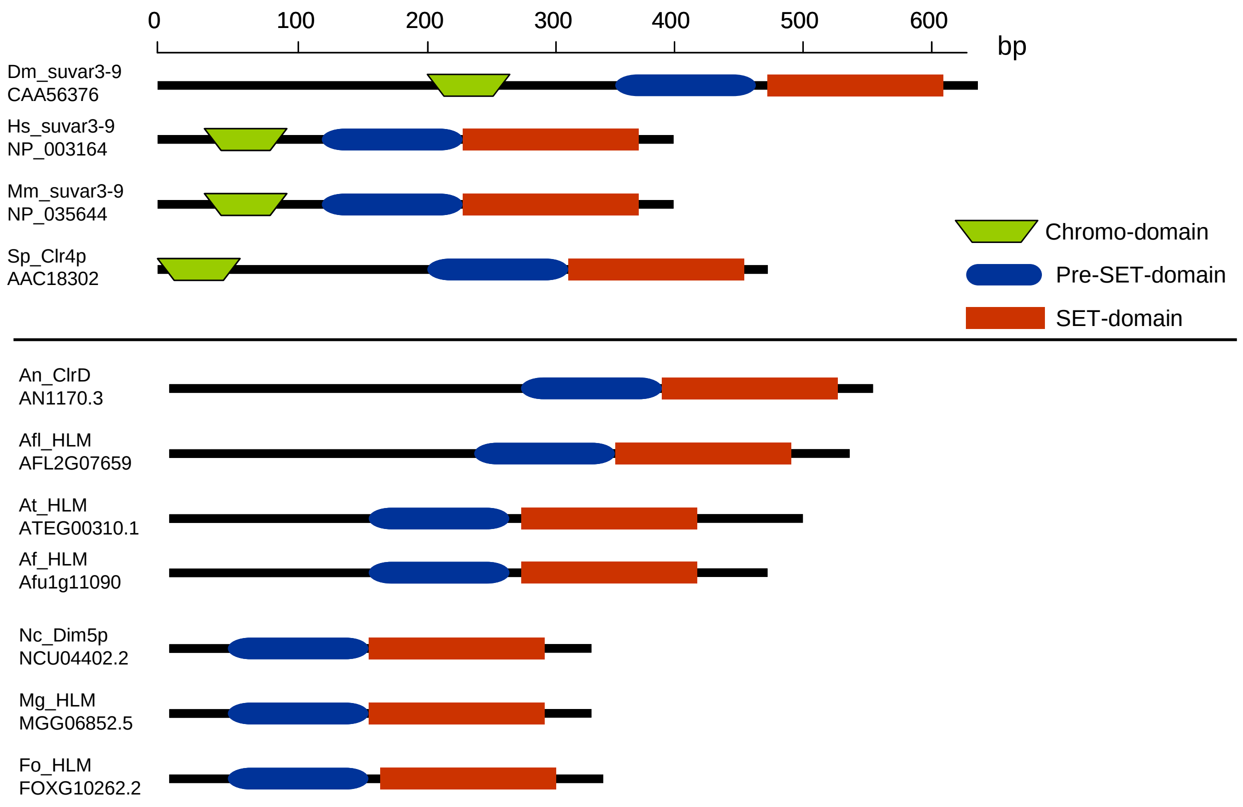

Supporting Figure S7. Domain organization of histone lysine methyltransferases (HLMs) homologous to su(var)3-9. With the exception of *S. pombe*, none of the fungal HLMs contain a chromo-domain typically found in the metazoan group; Dm: *Drosophila melanogaster*; Hs: *Homo sapiens*; Mm: *Mus musculus*; Sp: *Schizosaccharomyces pombe*; An: *A. nidulans*; Afl: *A. flavus*; At: *A. terreus*; Af: *A. fumigatus*; Nc: *Neurospora crassa*; Mg: *Magnaporthe oryzae*; Fo: *Fusarium oxysporum.* Fungal histone H3 K9 methyltransferase function was shown for *S. pombe* Clr4, *N. crassa* Dim5, and now, for *A. nidulans* ClrD. bp, base pairs.

Reyes et al., Supporting Figure S8

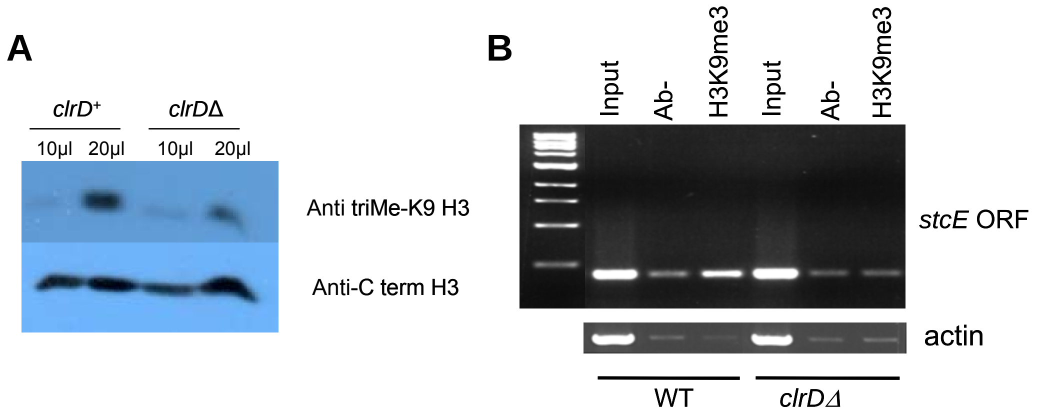

Supporting Figure S8. Western and ChIP analysis of histone H3 methylation in the *clrD*Δ deletion strain and the isogenic *clrD*$^+$ wild type control. **A.** Western analysis of nuclear extracts in *clrD*$^+$ and *clrD*Δ strains. From each strain, 10µl or 20µl of total protein extracts (0,5µg/µl) were separated by SDS-PAGE, blotted onto nitrocellulose and probed with the antibody recognizing H3K9me3 (Anti triMe-K9H3). The same membrane was stripped and re-probed with the H3-C-terminal antibody to determine the ratio between H3K9me3 and total H3 in the sample. **B.** ChIP analysis of the *stcE* open reading frame (ORF). The agarose gel (including DNA size ladder, first lane) shows amplification products of the *stcE* and actin genes after ChIP analysis using the H3K9me3 antibody shown in panel A. WT (corresponding to *clrD*$^+$) and *clrD*Δ strains were grown in liquid GMM for 36 hours at 37°C and processed as described in Methods. Input controls for both strains show strong amplification for *stcE* (*stcE* ORF) and actin (loading control). In the ChIP reactions actin signals are low, as expected. In control reactions omitting the antibody (Ab-) only weak amplification levels for *stcE* are seen. In the reactions adding the antibody (H3K9me3) amplification in the wild type is significantly higher than in the *clrD*Δ strain, which only shows the weak background H3K9me3 level (compare to lane Ab- in *clrD*Δ). This indicates that the *stcE* gene carries trimethylated lysine 9 at H3 and that ClrD is responsible for this histone modification.
